# Donanemab physiologically based pharmacokinetic model coupled with natural life cycle model for amyloid plaque aggregation to predict target CSF and ISF concentrations associated with amyloid plaque reduction

**DOI:** 10.1002/alz70856_103434

**Published:** 2025-12-25

**Authors:** Drew Butkowski, Ronald B. DeMattos, Sergey Shcherbinin, Dawn A. Brooks, John R. Sims, Ivelina Gueorguieva

**Affiliations:** ^1^ Eli Lilly, Indanapolis, IN, USA; ^2^ Eli Lilly and Company, Indianapolis, IN, USA; ^3^ Eli Lilly and Company, Bracknell, Berkshire, United Kingdom

## Abstract

**Background:**

Whole body physiologically based pharmacokinetic (PBPK) models describe the disposition of antibody therapeutics into the CNS and are used to simulate dynamics of cerebrospinal (CSF) and interstitial (ISF) fluid. These models may also predict the natural life cycle of amyloid aggregation and changes following treatment with amyloid targeting agents. Donanemab is an IgG1 antibody targeting N3pG (a modified beta‐amyloid present solely in brain plaques) indicated for treatment in early symptomatic AD patients. Use of donanemab PBPK model allows for identification of target ISF levels and impact on amyloid plaque level in the brain using different dosing regimens. The aim of this analysis is to establish donanemab PBPK/amyloid plaque model.

**Method:**

A longitudinal natural life cycle model of amyloid aggregation was established in early symptomatic AD participants. The impact on the amyloid aggregation following donanemab treatment and achieving the targeted ISF concentrations was evaluated. Analyses included participants with early symptomatic AD (mild cognitive impairment or mild‐dementia due to AD) in the combined population from a phase 1 study (*N* = 61; NCT02624778), phase 2 study (TRAILBLAZER‐ALZ; *N* = 256; NCT03367403) and phase 3 study (TRAILBLAZER‐ALZ2; *N* = 1954; NCT04437511).

**Result:**

Age and genotype‐related amyloid aggregation pathology was simulated to predict longitudinal changes in plasma Aβ_42_/ Aβ_40_ ratio, CSF Aβ_42_, ISF Aβ_42_ and amyloid plaque level in the brain. Around the age of 60 years there was a steep decline in CSF, ISF Aβ_42_ and plasma Aβ_42_/ Aβ_40_ ratio with APOE4 carriers commencing this decline slightly earlier than non‐carriers, but with similar rate. Correspondingly there is a steep transition around the same age for amyloid plaque level to increase followed then by a slow saturation at higher ages and with these changes starting at slightly earlier age for APOE4 carriers. Following donanemab treatment in early symptomatic AD participants with mean age of 73.2 years and 71% APOE4 carriers, amyloid reduction was associated with maintaining median serum donanemab concentrations over 15 µg/mL (95% confidence interval 8.54─18.0) with corresponding CSF and ISF levels, resulting in robust amyloid plaque reduction.

**Conclusion:**

Increased understanding of the disposition of amyloid‐targeting agents in the CNS allows for better understanding of ISF concentrations associated with therapeutic effect.

